# Soymilk yogurt prepared using *Pediococcus pentosaceus* TOKAI 759m ameliorates cognitive function through gut microbiota modulation in high-fat diet mice

**DOI:** 10.1016/j.crfs.2025.100993

**Published:** 2025-02-03

**Authors:** Yuki Nakashima, Tomoyuki Hibi, Masafumi Urakami, Maki Hoshino, Taiki Morii, Hikari Sugawa, Nana Katsuta, Yuki Tominaga, Himeno Takahashi, Asako Otomo, Shinji Hadano, Shin Yasuda, Ayaka Hokamura, Saki Imai, Hideki Kinoshita

**Affiliations:** a Graduate School of Bioscience, Tokai University, 871-12 Sugido, Mashiki-machi, Kamimashiki-gun, Kumamoto, Japan; b JSPS Research Fellowship for Young Scientists, Tokyo, 102-0083, Japan; c Research Institute of Agriculture, Tokai University, Kumamoto, 862-8652, Japan; d School of Agriculture, Tokai University, 871-12 Sugido, Mashiki-machi, Kamimashiki-gun, Kumamoto, Japan; e Graduate School of Agriculture, Tokai University, 871-12 Sugido, Mashiki-machi, Kamimashiki-gun, Kumamoto, Japan; f Molecular Neuropathobiology Laboratory, Department of Physiology, Tokai University School of Medicine, Isehara, Kanagawa, 259- 1193, Japan; g Probio Co., Ltd., 1330-1 Futa, Nishihara-mura, Aso-gun, Kumamoto, Japan

**Keywords:** Lactic acid bacteria, Soymilk yogurt, Cognitive function, Obesity, Anti-inflammation, Microglia, Gut microbiota

## Abstract

Recent studies have confirmed that obesity leads to neuroinflammation and cognitive decline. This study aimed to examine whether soymilk yogurt prepared using *Pediococcus pentosaceus* TOKAI 759m could prevent cognitive decline and neuroinflammation progression in mice fed a high-fat diet (HFD). C57BL/6NJcl male mice were grouped according to the following dietary interventions and monitored for 15 weeks: (1) normal control diet, (2) HFD, (3) HFD with soymilk (SM), (4) HFD with soymilk yogurt (SY), and (5) HFD with bacterial cells of the starter strain (BC). The levels of inflammatory cytokines in serum and hippocampus were measured. Compared to the HFD group, the SY group scored higher in the novel object recognition test and exhibited lower levels of Interleukin-6 (IL-6) and Tumor Necrosis Factor (TNF)-α in the hippocampus. However, the SM and BC groups did not show these significant changes. Proteomic analysis of the hippocampus revealed three enriched protein clusters in the SY group: synaptic proteins, glycolysis, and mitochondrial ATP formation. Fecal samples were also collected to measure the proportion of gut microbiota using 16S rRNA analysis. Interestingly, the proportion of butyrate-producing bacteria, such as *Clostridium* and *Akkermansia*, tended to be higher in the SY group than in the HFD group. An additional *in vitro* study revealed that the components of SY, such as daidzein, genistein, and adenine, could decrease inflammatory cytokine levels in microglial cells. In conclusion, soymilk yogurt prepared using *P. pentosaceus* TOKAI 759m may modulate gut microbiota and prevent neuroinflammation, thereby leading to a possible improvement in cognitive function.

## Highlights

• Soymilk yogurt ameliorated cognitive function in high-fat diet induced obesity mice.
• Soymilk yogurt suppressed inflammatory markers of the obesity mice.
• Proteomic analysis of the brain revealed 3 enriched clusters in soymilk yogurt group.
• Soymilk yogurt elevated the proportion of butyrate-producing bacteria in the gut.
• Some components of soymilk yogurt suppressed the production of cytokine in microglia.

## Graphical abstract

**Figure:**
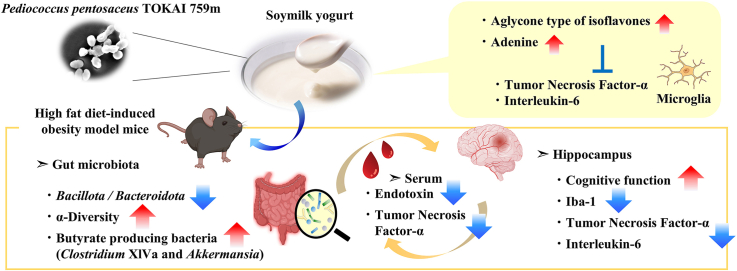

## 1 Introduction

Globally, about 57.4 million people were living with dementia in 2019, and this number is estimated to reach 152.8 million by 2050 (GBD 2019 Dementia Forecasting Collaborators, 2022). Preventing dementia is a global public health priority due to the enormous and growing societal cost of the condition (GBD 2019 Dementia Forecasting Collaborators, 2022). Neurodegenerative diseases, such as Alzheimer's disease and related dementias, have multiple etiologies; however, no curative treatment has been developed (Passeri et al., 2022). A greater understanding of the pathophysiology of dementia, including causative risk factors, will accelerate the development of new therapies (Wong Zhang et al., 2023).

Obesity is one risk factor of cognitive impairment and dementia, and it affected 107.7 million children and 603.7 million adults worldwide in 2015 (Afshin et al., 2017; O'Brien et al., 2017). Substantial research has indicated that a western diet accelerates and enhances inflammation and the induction of dementia, while dietary manipulations hold promise for dementia prevention and support in therapy (Więckowska-Gacek et al., 2021). An epidemiological investigation showed that overweight and abdominal obesity are associated with increased dementia incidence (Ma et al., 2020). Several animal studies have demonstrated that high-fat diet (HFD)-induced obesity is associated with deficits in hippocampus-dependent memory learning (Bocarsly et al., 2015; Pan et al., 2023). Habitual consumption of healthy foods has been shown to contribute to the maintenance and improvement of higher cognitive performance. Recent cohort studies have indicated that higher adherence to a Mediterranean diet is associated with lower dementia risk, underscoring the importance of diet in dementia prevention (Shannon et al., 2023).

Several studies have shown that food intake can alter the microbiome, potentially modulating brain function and behavior through the “gut–brain axis.” An HFD disturbs gut microbiota composition and increases inflammatory cytokine levels in the serum, gut, and brain (Jeong et al., 2019; Yang et al., 2019). HFD-induced obesity results in increased *Bacillota* and decreased *Bacteroidota* abundance, disrupting the gut microbiota; the ratio between *Bacteroidota* and *Bacillota* is crucial for weight loss (Leigh and Morris, 2020). Bruce-Keller et al. (2015) reported that cognitive behavior and cerebrovascular homeostasis were disrupted, along with increased neuroinflammation in HFD-fed mice. Furthermore, Fröhlich et al. (2016) demonstrated that treatment with antibiotics disturbs the gut microbiota and impairs object recognition in mice. Recent evidence indicates that the intake of probiotics or healthy foods prevents gut microbiota disruption and improves neuroinflammation and cognitive function (Yang et al., 2019; Shi et al., 2020; Duan et al., 2021; Lan et al., 2023).

Soy-related food has recently been expected to play a role in several health benefits, such as reducing cancer risk, alleviating menopausal symptoms, and improving memory function (Messina et al., 2022). Fermented soymilk yogurt (SY) prepared using lactic acid bacteria (LAB) contains numerous bioactive components, including aglycone isoflavones, saponins, peptides, free amino acids, vitamins, and minerals (Kumari et al., 2022; Nakashima et al., 2022). A previous study showed that dietary intervention with SY prepared using *Pediococcus pentosaceus* TOKAI 759m induced high antioxidant activity, reduced proinflammatory cytokine production, and altered the composition of the cecum microbiota (Yamamoto et al., 2019; Nakashima et al., 2024). Furthermore, SY prepared in this way contains high levels of aglycone-type isoflavones, including daidzein and genistein, as well as other beneficial components such as adenine and N1,N8-diacetylspermidine (Nakashima et al., 2024). Some studies have reported that individual probiotic strains (Yang et al., 2019; Zheng et al., 2024) or components of soymilk, such as soy isoflavones (López et al., 2018; Pang et al., 2020), play a potential role in reducing neuroinflammation and improving cognitive decline in HFD-induced obesity model mice; however, the effects of whole SY, including both the starter strain and soy components, have not been fully elucidated. Furthermore, it is unclear how the intake of these SY products influences gut microbiota and brain functions. In this study, we examined the potential effects of SY prepared using *P. pentosaceus* TOKAI 759m in preventing cognitive impairment and explored the underlying mechanisms of the microbiota–gut–brain axis in HFD-induced obesity model mice.

## 2 Materials and methods

### 2.1 Cell culture and reagents

MG6 mouse microglial cells (Riken BRC) were cultured in high-glucose Dulbecco's modified Eagle's medium (DMEM) (FUJIFILM Wako Pure Chemical Co., Osaka, Japan) containing 10% fetal bovine serum (FBS) supplemented with 10 μg/mL recombinant human insulin (FUJIFILM Wako Pure Chemical Co.), 0.1 mM 2-mercaptoethanol (FUJIFILM Wako Pure Chemical Co.), and 1% penicillin/streptomycin (FUJIFILM Wako Pure Chemical Co.) with 5% CO_2_ atmosphere in a humidified 37 °C incubator. Ninety-six half-area microplates (Greiner Bio-One, Kremsmünster, Austria) and enzyme-linked immunosorbent assay (ELISA) kits (ELISA MAX™ Deluxe Set Mouse, TNF-α: 430904 and IL-6: 431304; BioLegend, San Diego, CA, USA) were used for ELISA. Isoflurane was obtained from FUJIFILM Wako Pure Chemical Co.

### 2.2 Bacterial strains and culture conditions

The LAB strain *P. pentosaceus* TOKAI 759m (Yamamoto et al., 2019) was used as a starter strain for the SY. TOKAI 759m was cultured twice for 24 h at 37 °C in De Man, Rogosa, and Sharpe (MRS) broth (Difco Laboratories, Detroit, MI, USA) with 2% (v/v) inoculum and subsequently used for experiments.

### 2.3 Sample preparation of SY and bacterial cells

The cultured TOKAI 759m (2% (v/v)) in MRS broth was added to sterilized plain soymilk (Organic Soymilk, Tokyo Meiraku, Tokyo, Japan) (Table S1) and further incubated for 24 h at 37 °C. After this, 2% (v/v) of the cultured soymilk was inoculated into fresh plain soymilk, followed by culturing for 24 h at 37 °C. The resulting SY samples were then freeze-dried. LAB strains were cultured in MRS broth for 24 h at 37 °C, after which they were collected, washed, and suspended in water. In our previous study, we determined that an optical density at 600 nm (OD_600_) of 1.0 corresponded to an estimated bacterial concentration of 1.5 × 10^8^ cells/mL, as measured by direct counting using a bacteria counter for certain bacterial taxa (Kinoshita, 2024). Therefore, a spectrophotometer (Shimadzu, Kyoto, Japan) was used to adjust the bacterial suspension to an OD_600_ of 1.0. The cells were then freeze-dried and incorporated into the HFD mixture.

### 2.4 Animals

Five-week-old male C57BL/6NJcl mice were purchased from CLEA Japan (Tokyo, Japan) and housed in plastic cages (three mice per cage) under a 12 h light/dark cycle with free access to food (regular chow diet: CLEA Rodent Diet CE-2, CLEA Japan, Tokyo, Japan) and water. After acclimatization for 4 weeks, mice were randomly divided into five groups (n = 12/group): (1) the normal control diet (ND) group was fed a regular chow diet (CLEA Rodent Diet CE-2, 11.9% kcal from fat), (2) the high-fat diet (HFD) group was fed an HFD (HFD-60, 60.7% kcal from fat; Oriental Yeast Co., Ltd., Tokyo, Japan) (Tables S2 and S3), (3) the soymilk (SM) group was fed an HFD with 0.5% soymilk (w/w), (4) the SY group was fed an HFD with 0.5% SY (w/w) prepared using *P. pentosaceus* TOKAI 759m, and (5) the bacterial cell (BC) group was fed an HFD with added *P. pentosaceus* TOKAI 759m (2 × 10⁸ CFU/g, with the same bacterial cell amount as the SY group). These samples were placed in a feeding dish to maintain free-feeding conditions for the animals. The cages were covered to prevent sample contamination between adjacent cages. Body weight and food intake were measured weekly, and fecal samples were collected in disinfected plastic cages to prevent contamination. Cognitive behavioral tests were conducted after 11 weeks of intervention. At the end of 15 weeks, the mice were euthanized under isoflurane anesthesia, the serum was collected, and the brain tissue was immediately excised for immunostaining (right hippocampus) and protein analysis (left hippocampus). The specimens were stored at −80 °C. The isolated tissues were fixed with 4% paraformaldehyde for histological analysis.

### 2.5 Behavioral tests of cognitive function

Cognitive function was evaluated using Y-maze and novel object recognition (NOR) tests. The Y-maze test was performed in a three-arm horizontal maze made from gray plastic (40 cm long, 3 cm wide, 12 cm high walls), according to the methods of Jang et al. (2018). Mice were initially placed within one arm of the maze, and the number of arm entries was manually recorded for each mouse over an 8-min period. An actual alternation was defined as entering all three arms in consecutive choices. The maze arms were thoroughly cleaned between tasks to remove residual odors. Alternation (%) was calculated as follows: alternation (%) = [(number of alternations)/(number of total arm entries−2)] × 100.

The NOR test was performed according to the method described by Wang et al. (2018) and contained two phases: the acquisition phase and the trial phase. For the acquisition phase, the mice were placed in a gray plastic box (40 × 40 × 40 cm) and allowed to explore freely for 5 min. Two identical objects were then placed at the two corners of the area, and the mice were allowed to explore freely for a further 10 min. At the end of the object exploration, the mice were returned to their home cages. In the trial phase 24 h after the initial exposure, one of the objects was replaced with a novel object, after which the mice were transferred again to the area for 5 min of object exploration in the presence of one familiar (A) or novel (B) object. During the test, the time spent in the object area was measured using SMART software (version 2.0, Panlab, Spain). The time spent exploring the novel object (B) was expressed as the recognition index (RI), which was indicated as follows: RI (%) = tB/(tA + tB).

### 2.6 Protein isolation and measurement

Proteins were isolated from the left hippocampus using the EzRIPA lysis kit (ATTO, Tokyo, Japan). Tissues were lysed using EzRIPA buffer with protease and phosphatase inhibitors. The lysed samples were then centrifuged (14,000×*g*, 15 min, 4 °C). Then, the supernatants were collected, and the protein was measured using a Micro BCA Protein Assay kit (Thermo Fisher Scientific, Waltham, MA, USA).

### 2.7 Determination of cytokine levels using ELISA

Quantitative assessment of Interleukin (IL)-6 and Tumor Necrosis Factor (TNF)-α in the serum and protein extracts of the hippocampus was conducted using an ELISA kit (ELISA MAX™ Deluxe Set Mouse TNF-α and IL-6, BioLegend), according to the manufacturer's instructions. Briefly, 25 μL of Capture Antibody (1X) was coated onto each well and kept overnight at 4 °C. Subsequently, each well was washed thrice with Phosphate-buffered saline (PBS) containing 0.05% Tween 20 (washing buffer). The wells were blocked with 1% Bovine serum albumin (BSA) (Nacalai Tesque, Kyoto, Japan) in PBS for 2 h. After washing the wells thrice, 25 μL of sample was added and incubated for 1 h. Then, after washing the wells thrice, 25 μL of Detection Antibody (1X) was added and incubated for 1 h. The wells were washed thrice and incubated with Avidin-HRP (1X) for 30 min. Then, 25 μL of Substrate Solution was added, and the reaction was terminated with 25 μL of 1 M sulfuric acid. The absorbance was measured at 450 nm using a microtiter plate reader. The concentration of cytokines was expressed as pg antigen per μg protein.

### 2.8 Determination of endotoxin concentration in the serum

The concentration of circulating serum endotoxin was measured using a ToxinSensor Chromogenic LAL Endotoxin Assay Kit (GenScript, Piscataway, NJ, USA), according to the manufacturer's instructions.

### 2.9 Evaluation of hippocampal protein using Western blot

Hippocampal protein samples were heated at 95 °C for 5 min, loaded onto SDS-PAGE (15 mA, 80 min), and transferred onto PVDF membranes (9 V, 55 min). The membranes were blocked in 5% skim milk at 4 °C overnight. The membranes were then washed thrice with Tris-buffered saline containing 0.1% Tween 20 (TBST) and incubated with antibodies against Iba-1 (1:2000, anti-Iba1 antibody, Rabbit, 019–19741, FUJIFILM Wako Pure Chemical Corporation) and β-actin (1:2000, beta actin antibody, Rabbit, GTX109639, GeneTex, Irvine, CA, USA) at room temperature for 1 h. The membranes were washed with TBST and incubated with HRP-conjugated goat anti-rabbit IgG (H + L) (1:25000, SA00001-2, Proteintech) at room temperature for 1 h. Protein bands were observed using ECL prime solution (Cytiva, Uppsala, Sweden) and the WSE-6100 LuminoGraphⅠ(ATTO). The gray values of the images were analyzed using ImageJ software (https://imagej.net/ij/).

### 2.10 Immunofluorescence assay

Immunostaining analysis of the brain slices was performed using the right hippocampus according to Jeong et al. (2019) with slight modifications. Briefly, the right-side brains were sectioned at a thickness of 20 μm and washed thrice with PBS containing 0.1% Triton X-100. The sections were blocked with 1% BSA in PBS containing 0.1% Triton X-100 for 2 h at room temperature, washed thrice, and incubated with anti-Iba-1 (1:1000, Rabbit, 019–19741, FUJIFILM Wako Pure Chemical Corporation) primary antibody overnight at 4 °C. After washing the sections thrice, they were stained with secondary antibodies conjugated to Alexa Fluor 488 (1:400, Jackson ImmunoResearch, West Grove, PA, USA) for 2 h at room temperature. Nuclei were stained with 1 μg/mL of Hoechst 33342 (Dojindo Laboratories, Kumamoto, Japan), and the Vector TrueVIEW Autofluorescence Quenching Kit (Vector Laboratories, Burlingame, CA, USA) was used to remove autofluorescence. Immunostained sections were scanned using an all-in-one fluorescence microscope (BZ-X810; KEYENCE, Osaka, Japan).

### 2.11 Fecal microbiota analysis

To assess trends in the composition of the gut microbiota for each group, equal amounts of fecal samples from each group were mixed and stored at −80 °C until use in the experiments. The 16S rRNA gene amplicon sequencing was conducted according to our previously reported method (Nakashima et al., 2024). Firstly, DNA was extracted from the fecal samples, and polymerase chain reaction (PCR) was performed. Secondly, the V3–V4 regions of the 16S rRNA gene were PCR-amplified using the universal primers (341F: 5′-CCTACGGGAGGCAGCAG-3′ and 805R: 5′-GGACTACCAGGGTATCTAAT-3′). Sequencing was conducted using paired-end reads with a 600 bp cycle on a MiSeq sequencing system, utilizing the MiSeq Reagent Kit ver.3 (Illumina, Inc., San Diego, CA, USA). Thirdly, we used Fastq-join to join the paired-end reads for each sample and subjected them to quality filtering using the FASTX Toolkit. After checking the sequence quality, the approved sequences were clustered into operational taxonomic units with 97% pairwise identities. Each representative tag was assigned to a taxon using RDP Classifier ver.2.13 (TechnoSuruga Laboratory, Shizuoka, Japan).

### 2.12 Sample preparation for liquid chromatography-mass spectrometry/mass spectrometry (LC-MS/MS)

Extracted hippocampal proteins from each group were mixed, further purified by methanol–chloroform precipitation, and solubilized in a phase-transfer surfactant buffer (Masuda et al., 2008). A total of 20 μg of protein was collected into a new tube and boiled (95 °C, 5 min). The protein was reduced with 10 mM TCEP, alkylated with 20 mM iodoacetamide, and purified using the protocol of SP3 (Hughes et al., 2019). Trypsin (protein weight: 1/50) and Lys-C (protein weight: 1/50) were added to digest the protein, followed by incubation for 16 h at 37 °C. Then, the collected peptides were desalted and purified using C18 StageTips. The purified samples were dried with SpeedVac and solubilized in 0.1% formic acid/2% acetonitrile.

### 2.13 Hippocampal protein analysis by LC-MS/MS

LC-MS/MS was performed by coupling an UltiMate 3000 Nano-LC system (Thermo Scientific, Bremen, Germany) and an HTC-PAL autosampler (CTC Analytics, Zwingen, Switzerland) with a trap column (0.075 × 20 mm, Acclaim PepMap RSLC Nano-Trap Column; Thermo Fisher Scientific) for sample injection and a nano-LC-MS interface (AMR, Tokyo, Japan) that ionized peptides using nano-electrospray ionization in positive-ion mode to a Q Exactive hybrid quadrupole-Orbitrap mass spectrometer (Thermo Scientific) using data-independent acquisition mode (DIA). Peptides were analyzed using an analytical column (75 μm × 30 cm) packed in-house with ReproSil-Pur C18-AQ resin (1.9 μm, Ammerbuch, Germany) at a flow rate of 280 nL/min and a 105-min gradient from 5% to 30% of solvent B (solvent A, 0.1% FA and 2% acetonitrile; solvent B, 0.1% FA and 90% acetonitrile). Mass spectra were acquired in the range of 398–802 m/z with a resolution of 140,000, an AGC target of 3 × 10^6^, a maximum injection time of 200 ms, and a normalized collision energy of 27.

The MS2 spectra were collected with the following parameters: an 8 *m*/*z* isolation window at a resolution of 70,000, an AGC target of 3 × 10^6^ ions, a maximum injection time of “auto” (for overlapping window patterns), and a normalized collision energy of 27. Raw MS data were processed using DIA-NN software (Ver. 1.7.12). The database search included all entries from the *Homo sapiens* UniProt database and a contaminant database (Tyanova et al., 2016). The MaxQuant computational platform for mass spectrometry-based shotgun proteomics was used. This database was concatenated with a reversed version of all protein sequences. The search parameters were as follows: up to two missed cleavage sites, peptide lengths of 7–30 residues, carbamidomethylation of cysteine residues (+57.021 Da) as a static modification, protein names from FASTA files for implicit protein grouping, robust liquid chromatography with high precision for the quantification strategy, and retention time-dependent for cross-run normalization. Precursor ions were filtered to a 1% false discovery rate. The obtained data were used for protein–protein interaction network and gene ontology analyses using the STRING database (version 12.0, https://string-db.org/).

### 2.14 Statistical analyses

The statistical significance of the differences between the ND or HFD group and the other groups was obtained by Dunnett's test using IBM SPSS Statistics software ver. 22 (IBM Corp., Armonk, NY, USA). Spearman's correlation coefficients were used to determine the relationships between behavioral tests, immunological function, and the relative abundance of fecal microbiota of the identified genera. Statistical significance was considered at a *P*-value <0.05.

## 3 Results

### 3.1 SY ameliorates cognitive impairment in HFD-induced obese mice

The experimental scheme is shown in Fig. 1A. The body weight of mice in the HFD group after 15 weeks was significantly higher than that of mice in the ND group (*P* < 0.001). There was no difference in weight gain between the HFD groups during the study period (Fig. 1B and C).

**Fig. 1:**
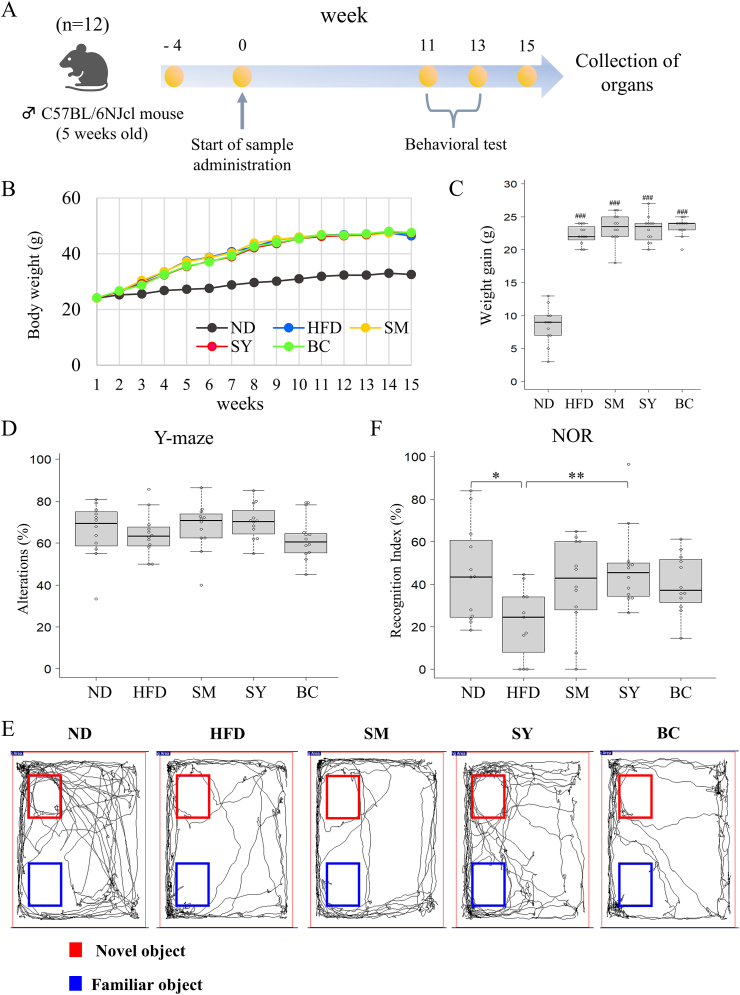
Effects of SY on body weight and cognitive function in HFD-induced obese mice. (A) Overview of the experimental timeline. (B) Changes in body weight. (C) Weight gain. (D) Spontaneous alternation behavior in the Y-maze test. (E and F) Tracking images and percentage of time spent with the novel object compared to the total object exploration time in the NOR test. The percentage of time spent with the novel object to total object exploration time is shown as RI (%). Results are expressed as the mean ± SD (n = 12). ^###^*P* < 0.001 vs. ND group. ∗*P* < 0.05, ∗∗*P* < 0.01 vs. HFD group. SY, soymilk yogurt (as fermented soymilk); HFD, high-fat diet; ND, normal control diet; SM, soymilk; BC, bacterial cell of starter strain; NOR, novel object recognition; RI, recognition index.

HFD feeding causes neuroinflammation and deficits in hippocampus-dependent memory (Jeong et al., 2019). To evaluate the effect of SY on cognitive decline induced by the HFD, we performed Y-maze and NOR tests as models reflecting hippocampus-dependent recognition memory. In the Y-maze test, alteration levels in the HFD group tended to be slightly lower than those in the ND group, whereas those in the SM and SY groups were higher than those in the HFD group (Fig. 1D). However, there were no significant differences among the five groups. In the NOR test, the percentage of time spent with the object in a novel location, defined as the RI, of the HFD group was significantly lower than that of the ND group (*P* < 0.05); however, the SY group showed a significant increase in RI compared to the HFD group (*P* < 0.01). No significant differences were observed among the ND, SM, and BC groups (Fig. 1E and F).

### 3.2 SY suppresses inflammation in serum and hippocampus

**Fig. 2:**
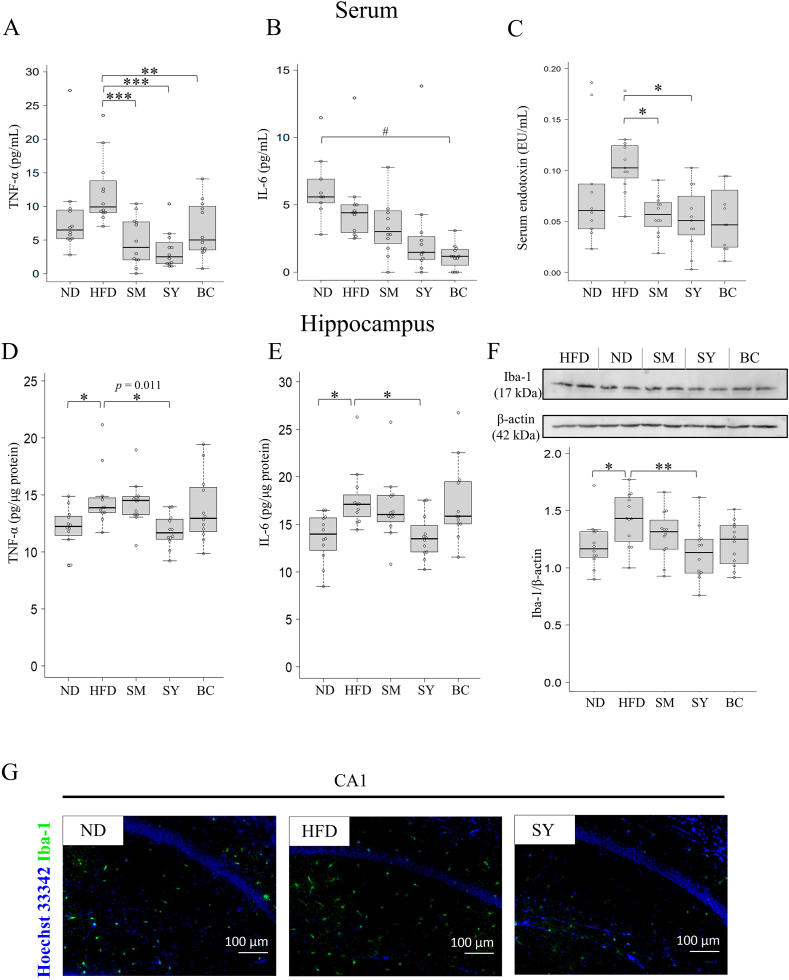
SY suppressed inflammation in the serum and hippocampus. The serum cytokine levels of (A) TNF-α (n = 12) and (B) interleukin (IL)-6 (n = 9–12). (C) Serum endotoxin levels (EU/mL, n = 10–12). Hippocampus cytokine levels of (D) TNF-α and (E) IL-6 (n = 11–12). (F) Representative Western blot images (top) of the protein levels of Iba-1 and β-actin (n = 2), with the quantified Iba-1/β-actin ratios (bottom) in the hippocampi of the groups (n = 12). (G) Immunofluorescent staining of Iba1 in CA1 of the hippocampus (scale bar: 100 μm). The results are expressed as the mean ± SD. ∗*P* < 0.05, ∗∗*P* < 0.01, ∗∗∗*P* < 0.001 vs. HFD group. SY, soymilk yogurt (as fermented soymilk); HFD, high-fat diet; ND, normal control diet; SM, soymilk; BC, bacterial cell of starter strain.

Neuroinflammation is a characteristic of cognitive deficits during which the activation of microglia has been implicated. The levels of inflammatory cytokines in the serum and hippocampus were measured using ELISA. The serum TNF-α levels significantly decreased in the SM, SY (*P* < 0.001), and BC (*P* < 0.01) groups compared to those in the HFD group (Fig. 2A). In contrast, the basal serum IL-6 levels tended to be higher in the ND group, while the levels in the SM, SY, and BC groups were lower than those in the HFD group (Fig. 2B). Furthermore, endotoxin levels in the serum of the SM and SY groups were significantly lower than those in the serum of the HFD group (*P* < 0.05) (Fig. 2C).

We evaluated the levels of these inflammatory cytokines in the hippocampus. The levels of both TNF-α and IL-6 increased in the HFD group compared to those in the ND group, while the SY group exhibited decreased levels of these cytokines compared to the HFD group (*P* < 0.05) (Fig. 2D and E). The expression of Iba-1 in the hippocampus was evaluated using western blotting (Fig. 2F). The levels of Iba-1, a marker of activated microglia, were significantly higher in the HFD group than in the ND group (*P* < 0.05). However, the SY group showed a significant decrease in Iba-1 levels compared to the HFD group (*P* < 0.01). Similarly, immunohistochemistry revealed that the distribution of Iba-1 in the tissues of the HFD group was higher than that of the ND group, whereas that of the SY group was lower than that of the HFD group (Fig. 2G).

### 3.3 Comprehensive analysis of hippocampal proteins by proteome analysis

**Fig. 3:**
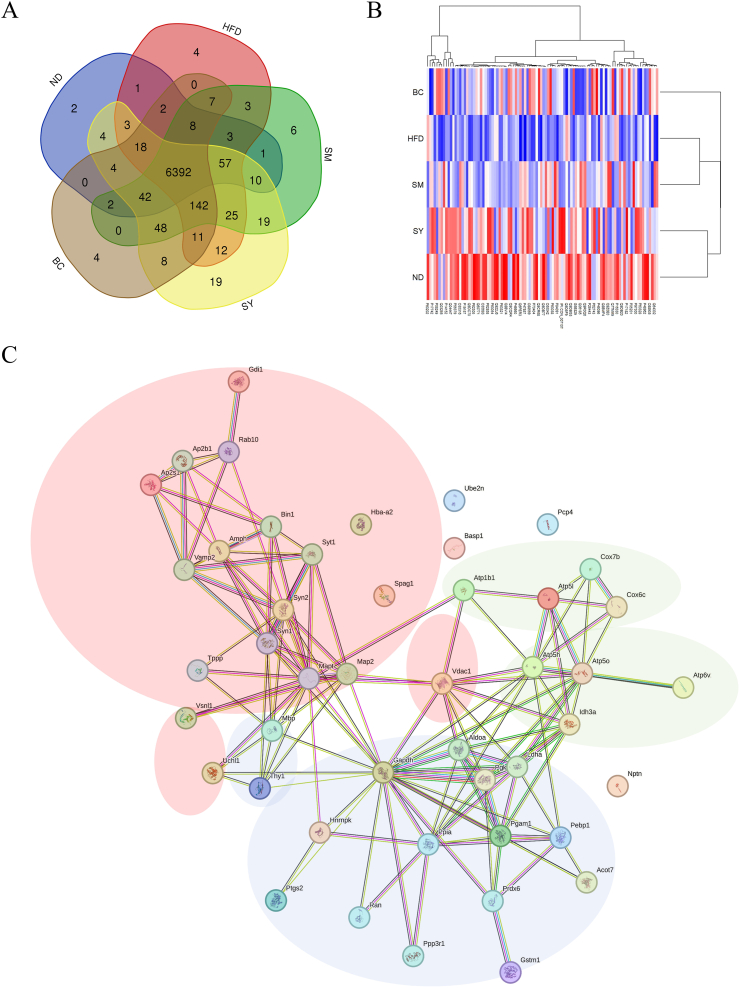
Comprehensive analysis of hippocampal proteins by proteome analysis. Venn diagram (A) and (B) heatmap using clustering analysis of determined hippocampus proteins in ND, HFD, SM, SY, and BC groups. (C) STRING protein–protein interaction network showing direct interactions among the top 100 proteins with the largest differences in log_2_ intensity values (SY-HFD). Cluster 1: synaptic proteins, such as SYN, MBP, and MAP (red circle in the top-left). Cluster 2: glycolysis proteins, such as GAPDH and phosphoglycerate kinase (blue circle in the bottom-middle). Cluster 3: proteins related to mitochondrial ATP formation, such as ATP synthase and cytochrome *c* oxidase (green circles in the right-middle). SY, soymilk yogurt (as fermented soymilk); HFD, high-fat diet; ND, normal control diet; SM, soymilk; BC, bacterial cell of starter strain.

Since the SY group exhibited attenuated hippocampal inflammation and cognitive decline, we hypothesized that changes in hippocampal proteins might contribute to the enhanced cognitive function observed in this group. LC-MS/MS was performed using a Q Exactive mass spectrometer. The Venn diagram in Fig. 3A shows the number of common proteins found in the hippocampus of each group. We identified 6392 of the 6857 common proteins in each group. Furthermore, we assessed the proteomic profiles of hippocampal proteins using clustering analysis. Fig. 3B shows a heatmap of the difference in the log_2_ intensity values (ND-HFD) of the top 100 proteins. The results were classified into three clusters comprising the ND and SY groups, HFD and SM groups, and BC group, respectively. We then conducted a network analysis for protein–protein interactions using the STRING database (Version 12.0). The top 100 proteins with differences in log_2_ intensity values (SY-HFD) were used to analyze protein–protein interactions using STRING. The results revealed three protein clusters with increased abundance in the SY group: (1) synaptic proteins, such as synapsin (SYN), myelin basic protein (MBP), and microtubule-associated protein (MAP) (see red circle in the top-left of the panel); (2) glycolysis proteins, such as GAPDH and phosphoglycerate kinase (see blue circle, bottom-middle); and (3) proteins related to the formation of ATP in mitochondria, such as ATP synthase and cytochrome *c* oxidase (see green circles, right-middle) (Fig. 3C). Table S4 shows the results of the gene ontology analysis, which predicted the pathways associated with these clusters.

### 3.4 SY alters the composition of gut microbiota in HFD mice

**Fig. 4:**
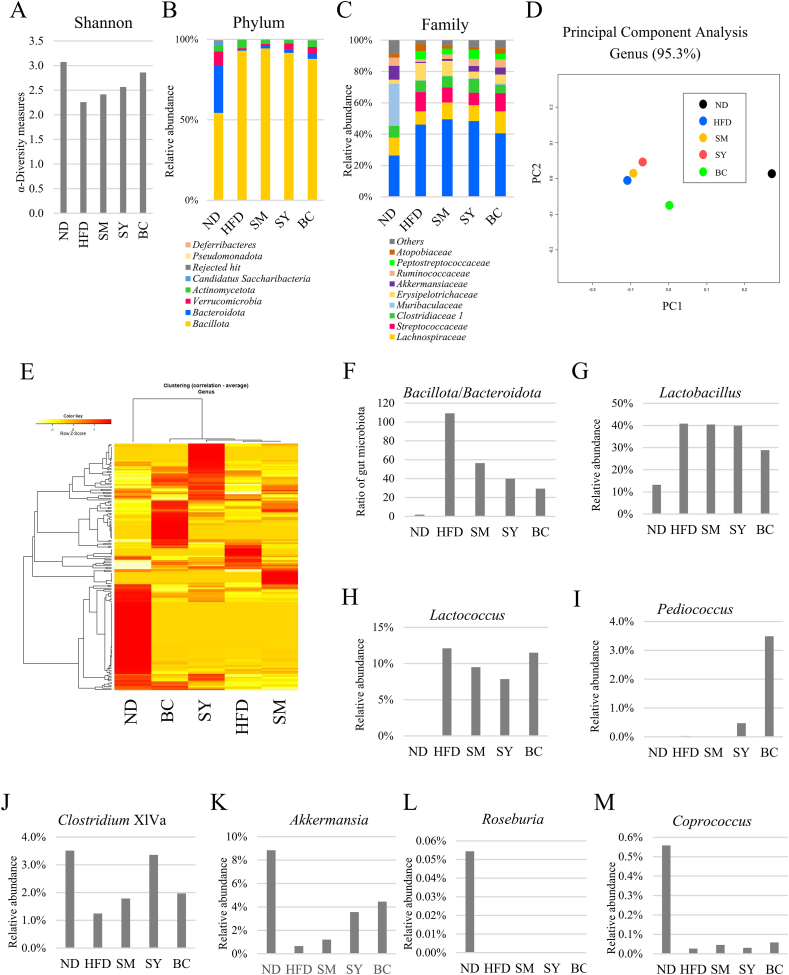
Effect of SY consumption on gut microbial structure in HFD-induced obese mice. (A) Alpha diversity using Shannon indices. (B, C) Proportion of microbiota at the phylum and family levels. (D) Principal component analysis. (E) Heatmap and clustering analysis of genera. (F) The ratio of *Bacillota*/*Bacteroidota*. Relative abundance of (G) *Lactobacillus*, (H) *Lactococcus*, (I) *Pediococcus*, (J) *Clostridium* XlVa, (K) *Akkermansia*, (L) *Roseburia*, and (M) *Coprococcus*. SY, soymilk yogurt (as fermented soymilk); HFD, high-fat diet; ND, normal control diet; SM, soymilk; BC, bacterial cell of starter strain.

We hypothesized that SY alters the composition of gut microbiota and contributes to the improvement of hippocampal function. Subsequently, we assessed the proportion of the gut microbiota using 16S rRNA gene amplicon sequencing. As shown in Fig. 4A, the Shannon indices of the ND, HFD, SM, SY, and BC groups were 3.07, 2.26, 2.42, 2.57, and 2.86, respectively. In particular, the SY and BC groups tended to show higher scores than the HFD group. At the phylum level, the HFD reduced the proportions of *Bacteroidota* and increased the proportions of *Bacillota* compared to the ND group, whereas the bacterial composition of the SM, SY, and BC groups seemed to show a slight improvement compared to that of the HFD group (Fig. 4B). At the family level, the HFD group showed attenuated proportions of *Muribaculaceae* and *Akkermansiaceae*, along with elevated proportions of *Lactobacillaceae*, *Streptococcaceae*, and *Erysipelotrichaceae,* compared to the ND group (Fig. 4C). Among the sample-fed groups, the proportions of these bacteria increased in the SY and BC groups. Moreover, principal component analysis (PCA) at the genus level, based on the distances between PCA plots, showed that the microbiota composition of the ND group was highly distinct from that of the HFD group (Fig. 4D). Additionally, the SY and BC groups tended to have similar distances from the ND plot compared to the HFD group. Heatmap clustering analysis of the fecal microbiota at the genus level (Fig. 4E) showed consistent patterns in the PCA. Furthermore, the ratio of *Bacillota*/*Bacteroidota* increased in the HFD group, whereas it tended to decrease in the SM, SY, and BC groups compared to the HFD group (Fig. 4F). At the genus level, the abundance of *Lactobacillus* and *Lactococcus* tended to be higher in the HFD, SM, SY, and BC groups than in the ND group (Fig. 4G and H). In contrast, the SY and BC groups tended to contain a higher percentage of *Pediococcus* than the ND and HFD groups (Fig. 4I). These results are in line with our experimental design using *Pediococcus pentosaceus* TOKAI 759m. We then evaluated the changes in butyrate-producing bacteria. *Clostridium* XlVa and *Akkermansia* tended to be lower in the HFD group than in the ND group, whereas those in the SY and BC groups showed an increasing trend compared to the HFD group (Fig. 4J and K). However, the abundance of *Roseburia* and *Coprococcus* was lower in the HFD, SM, SY, and BC groups than in the ND group (Fig. 4L and M).

### 3.5 Correlation between inflammation, memory function, and gut microbiota

**Fig. 5:**
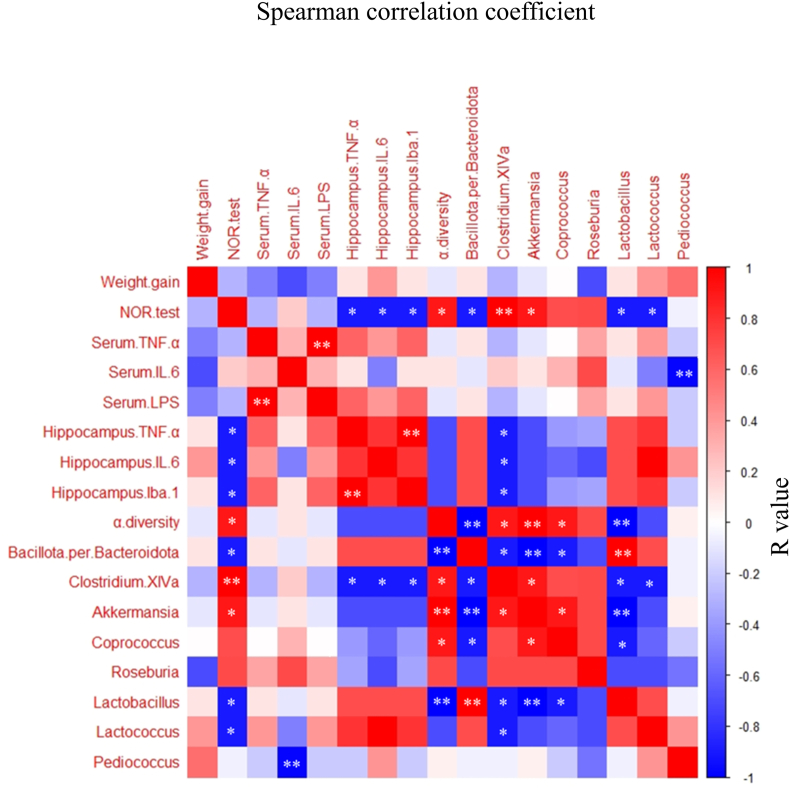
Spearman's correlation heatmap analysis of behavior tests, immunological function, and fecal microbiota of the identified genera. Red color indicates a positive correlation, and blue indicates a negative correlation (∗*P* < 0.05, ∗∗*P* < 0.01). SY, soymilk yogurt (as fermented soymilk); HFD, high-fat diet; ND, normal control diet; SM, soymilk; BC, bacterial cell of starter strain.

The correlation between behavioral tests, immunological function, and the relative abundance of fecal microbiota was determined using Spearman's correlation analysis (Fig. 5). NOR test scores were negatively correlated with the expression of the hippocampal inflammation markers TNF-α, IL-6, and Iba-1 (r = −0.9, *P* < 0.05). However, NOR test scores showed a positive correlation with α-diversity (r = 0.9, *P* < 0.05), *Clostridium* XlVa (r = 1.0, *P* < 0.01), and *Akkermansia* (r = 0.9, *P* < 0.05) and a negative correlation with the *Bacillota*/*Bacteroidota* ratio and the abundance of *Lactobacillus* and *Lactococcus* (r = −0.9, *P* < 0.05).

### 3.6 Suppressive effects of several representative SY components on proinflammatory cytokine production in LPS-stimulated microglia cells *in vitro*

**Fig. 6:**
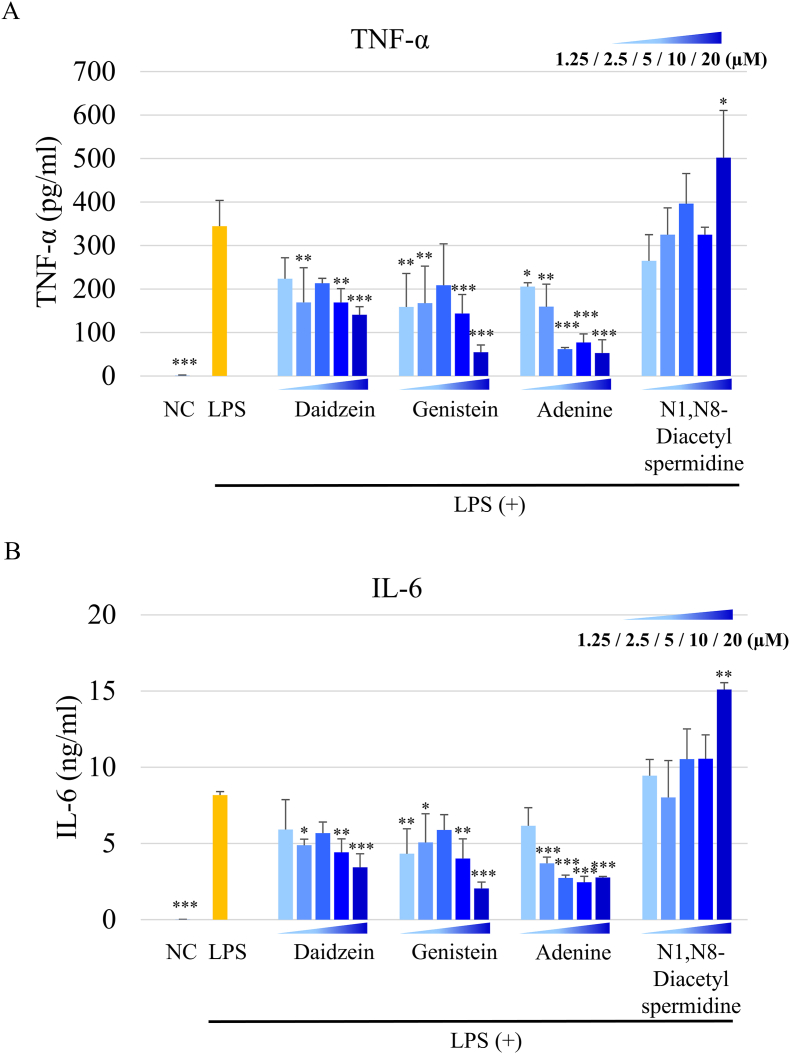
Assessment of anti-inflammatory activity using components of SY in LPS-stimulated MG6 cells. Cells were seeded at 5 × 10^4^ cells/well in a 96-well plate and pre-incubated for 2 h. Then, the cells were treated with 10 μL/well of samples (final concentration: 1.25–20 μM). After 24 h of incubation, the cells were stimulated with 10 μL/well of LPS (final concentration: 10 ng/mL) for 24 h. The supernatants were collected, followed by measuring the concentration of (A) TNF-α and (B) IL-6 using ELISA. Results are expressed as the mean ± SD (n = 3). ∗*P* < 0.05, ∗∗*P* < 0.01, ∗∗∗*P* < 0.001 vs. LPS group in the absence of samples. LPS, lipopolysaccharide; ELISA, enzyme-linked immunosorbent assay; SY, soymilk yogurt (as fermented soymilk).

In our previous study, we determined that SY prepared with *P. pentosaceus* TOKAI 759m had a high content of daidzein, genistein, adenine, and N1,N8-diacetylspermidine (Nakashima et al., 2024). Furthermore, we revealed that some of these components contribute to anti-inflammatory effects in an LPS-induced macrophage cell line (Nakashima et al., 2024). Here, we determined the anti-inflammatory activity of several representative components present in SY using LPS-induced MG6 microglia cells as an *in vitro* cell model. Before the ELISA experiments, sample concentrations were assessed for cytotoxicity using the WST-8 assay to ensure non-toxic levels (Fig. S1). Fig. 6A and B shows the concentration of TNF-α and IL-6 in the supernatant of LPS-stimulated MG6 cells, respectively. LPS significantly increased the levels of both cytokines compared with the negative control group (*P* < 0.01), whereas daidzein, genistein, and adenine significantly decreased the levels of these cytokines upon LPS stimulation (*P* < 0.05).

## 4 Discussion

There is a concern that a western high-fat and/or high-caloric diet can easily lead to obesity, brain inflammation, and induce dementia (López-Taboada et al., 2020; Więckowska-Gacek et al., 2021). Furthermore, HFD-induced obesity can alter the structure of gut microbiota and affect the serum LPS levels, along with disruption of tight junctions in the intestinal barrier in model mice (Jeong et al., 2019). Here, we demonstrate that SY prepared using *P. pentosaceus* TOKAI 759m can prevent inflammation and attenuate cognitive function decline in HFD-induced mice. Moreover, we examined gut microbiota composition and explored the components with anti-inflammatory activity in SY.

We first investigated changes in weight gain among the experimental groups of mice. Animals in the HFD-fed groups were significantly heavier than those in the ND group (Fig. 1B and C), whereas serum endotoxin levels were lower in the SM and SY groups than in the HFD group (Fig. 2C). Furthermore, we observed lower serum levels of TNF-α, a major proinflammatory cytokine, in the SM, SY, and BC groups compared to the HFD group (Fig. 2A). In contrast, serum IL-6 levels in the ND group tended to be higher than those in the HFD group (Fig. 2B). These results suggest that IL-6 functions not only in inflammation but also in modulating feeding and improving glucose tolerance (Timper et al., 2017). Some studies have reported that HFD-induced obesity leads to elevated gut microbiota-generated LPS in the circulation, which promotes proinflammatory cytokine production (Hersoug et al., 2018). Our results suggest that SY suppresses the production of inflammatory cytokines by inhibiting the increase in LPS in the blood.

Microglia are resident monocyte-lineage cells in the brain and play a significant role in the pathological and regenerative states of the brain (Nakajima and Kohsaka, 2001). It has been reported that bacterial LPS migrates into the circulation and acts as an endotoxin on microglia, inducing neuroinflammation and cognitive impairment in HFD-induced obese mice (Jeong et al., 2019). In our results, the expression of Iba-1, a marker of activated microglia, was downregulated by SY (Fig. 2F and G). Moreover, the SY group exhibited decreased levels of TNF-α and IL-6 in the hippocampus compared to the HFD group (Fig. 2D and E). Furthermore, in the NOR test, the RI (%) was lower in the HFD group than in the ND group, while the SY group had a higher score than the HFD group (Fig. 1E). Although no significant differences were observed in the SY group's working memory performance in the Y-maze test, a trend toward improvement was noted (Fig. 1D), suggesting that supplementing SY with TOKAI 759m may contribute to maintaining brain function. These results suggest that the reduction in endotoxin translocation by SY might be important for attenuating the inflammatory cascade in the hippocampus and alleviating cognitive decline. Furthermore, the improvement in cognitive function with SY is most likely due to the suppression of inflammatory cytokines and not weight loss. In our previous study, we found that SY has a high content of compounds such as daidzein, genistein, adenine, and N1,N8-diacetylspermidine through metabolite analysis. Importantly, we observed the anti-inflammatory activity of aglycone isoflavones and adenine in an LPS-induced macrophage cell line (Nakashima et al., 2024). It has been reported that daidzein and genistein can inhibit the activation of the LPS-induced BV2 microglia cell line (Subedi et al., 2017; Wu et al., 2018). However, the anti-inflammatory activity of adenine and N1,N8-diacetylspermidine in microglia cells remains unclear. Thus, we evaluated the production of inflammatory cytokines in LPS-induced MG6 microglial cells and found significant inhibition of daidzein, genistein, and adenine (Fig. 6).

Long-term intake of an HFD accelerates cognitive decline and neuroinflammation and attenuates neuronal activity. Some studies have shown a disruption of synaptic-related proteins, such as MBP, MAP2, ATP synthase, and cytochrome *c* oxidase. Paul et al. (2024) showed a decline in MBP in the brains of HFD mice. Furthermore, McLean et al. (2019) indicated that a disturbance in MAP, which has cytoskeletal functions in neurons, can affect synaptogenesis and cognitive function in HFD mice. Moreover, Wen et al. (2023) reported that an HFD induces mitochondrial dysfunction, inhibits mitophagy, and can cause synaptic damage, eventually leading to cognitive decline. Interestingly, our results of the hippocampal proteome analysis showed that SY prevented the decline in these proteins. However, the extent to which differences in peak area values in proteome analysis affect brain function needs to be evaluated in detail in future studies. Recently, it has been reported that some components of SY prepared using *P. pentosaceus* TOKAI 759m may protect neuronal activity. Several studies have shown that daidzein, genistein, and their metabolites contribute to protecting neurons against oxidative stress, amyloid-beta-induced toxicity in model neuron cells, and HFD-induced obesity in model mice (López-Taboada et al., 2020; Pang et al., 2020; Petry et al., 2020; Ito et al., 2023). Additionally, Li et al. (2021) demonstrated that soy isoflavones can protect neurons against atrazine-induced toxicity by activating mitophagy through the BEX2/BNIP3/NIX signaling pathway *in vitro*. Moreover, Yoshimi et al. (2003) found that adenine exerts neurotrophic effects in Purkinje cells; however, the detailed mechanism remains unknown. Previously, we measured the concentration of aglycone isoflavones in SY prepared with *P. pentpsaceus* TOKAI 759m and reported levels of 27.5 ± 1.6 μM for daidzein and 32.7 ± 2.2 μM for genistein (Nakashima et al., 2024). Additionally, we observed that gut microbiota can convert glycoside isoflavones into aglycon isoflavones and glycosides. Kaneko et al. (2014) reported that 100 g of SM contained 7.7 mg of adenine. We did not determine the adenine levels in SY prepared with *P. pentpsaceus* TOKAI 759m. Therefore, it will be necessary to measure the concentrations of these components in the mouse brain in future studies.

The HFD leads to an increase in *Bacillota* and a decrease in *Bacteroidota* abundance, causing a disruption in gut microbiota (Leigh and Morris, 2020). Recent studies suggest that the balance of gut microbiota is crucial for brain function. Jeong et al. (2019) reported that an HFD induces dysbiosis among gut microbiota, with particular increases in the ratios of *Bacillota* to *Bacteroidota* and *Pseudomonadota* to *Bacteroidota*. Furthermore, Yang et al. (2019) demonstrated that transplantation of HFD microbiota confers hippocampus-dependent learning and memory deficits in normal mice, whereas oral treatment with *Akkermansia muciniphila* reduced hippocampal inflammation, restored neuronal development, and ameliorated defects in learning and memory in HFD mice. Zheng et al. (2024) reported that oral supplementation with the probiotic *Clostridium butyricum* improved hippocampal synaptic ultrastructure and ameliorated cognitive impairment in HFD mice. Furthermore, it has been suggested that these intestinal bacteria ameliorate the disruption of intestinal tight junctions and prevent the entry of LPS into the serum (Yang et al., 2019; Zheng et al., 2024). In our study, Spearman's correlation analysis (Fig. 5) revealed that the NOR test scores showed a significantly negative correlation with hippocampal inflammatory markers (TNF-α, IL-6, and Iba-1) and the ratio of *Bacillota*/*Bacteroidota* in fecal microbiota. In contrast, the scores showed a significantly positive correlation with α-diversity and the proportion of some butyrate-producing bacteria, such as *Clostridium* XlVa and *Akkermansia*. Several studies have shown that butyrate has anti-inflammatory potential and improves cognitive memory. Fernando et al. (2020) reported that sodium butyrate treatment reduces Aβ levels and improves cognitive function in Alzheimer's disease transgenic mice. Furthermore, Duan et al. (2021) demonstrated that butyrate treatment inhibits neuroinflammation, improves dendritic spine loss and social deficits, and improves anxiety-like behavior in HFD-fed mice. These results indicate that the anti-inflammatory activities in the hippocampus and the amelioration of cognitive decline may be attributed not only to the compounds present in SY but also to the modulation of gut microbiota. In addition, since equal amounts of fecal samples were pooled for 16S rRNA analysis in each group in this study, future studies should evaluate individual detailed differences.

## 5 Conclusion

In this study, we revealed the potential usefulness of SY prepared using *P. pentosaceus* TOKAI 759m to reduce cognitive decline in an HFD-induced obese mouse model. Importantly, SY contains high levels of aglycone isoflavones and adenine, and these major components may play a role in inhibiting inflammation in activated microglial cells, as demonstrated *in vitro*. SY did not cause weight loss but partially modulated the disruption of gut microbiota, thereby contributing to the maintenance of synaptic function and reducing inflammatory cytokine production. This is the first report to demonstrate anti-inflammatory effects and attenuated cognitive decline in obese mice using SY, as opposed to using only starter LAB or cow's milk-based yogurt.

The limitations of this study include the need to determine the extent to which SY components are transferred to the brain and to elucidate the mechanisms by which changes in the intestinal microbiota influence this process. One limitation of this study is that the gut microbiota and brain proteome analyses show overall trends with samples pooled in each group rather than individual data. Therefore, future studies will need to compare each of these individually to elucidate the mechanism in more detail. Further studies aimed at clarifying these findings may contribute to preventing obesity-related brain inflammation and cognitive decline through the intake of SY.

## CRediT authorship contribution statement

**Yuki Nakashima:** Conceptualization, Writing – original draft, Writing – review & editing, Investigation, Visualization, Formal analysis. **Tomoyuki Hibi:** Conceptualization, Investigation, Formal analysis. **Masafumi Urakami:** Investigation, Visualization, Formal analysis. **Maki Hoshino:** Investigation, Formal analysis. **Taiki Morii:** Investigation, Visualization. **Hikari Sugawa:** Investigation, Methodology. **Nana Katsuta:** Investigation, Methodology. **Yuki Tominaga:** Investigation. **Himeno Takahashi:** Investigation. **Asako Otomo:** Writing – review & editing, Conceptualization, Supervision, Methodology. **Shinji Hadano:** Writing – review & editing, Conceptualization, Supervision, Methodology. **Shin Yasuda:** Writing – review & editing, Supervision, Methodology. **Ayaka Hokamura:** Supervision, Methodology. **Saki Imai:** Conceptualization, Supervision, Methodology. **Hideki Kinoshita:** Writing – original draft, Writing – review & editing, Conceptualization, Supervision.

## Ethics statement

Experiments were performed in accordance with the guidelines of the Institutional Animal Ethics Committee of Tokai University (approval number: 222043).

## Declaration of competing interest

Hideki Kinoshita is the manager at Probio Co., Ltd. The authors declare that the research was conducted in the absence of commercial or financial relationships that could be construed as potential conflicts of interest.

## Figures and Tables

**figs1:**
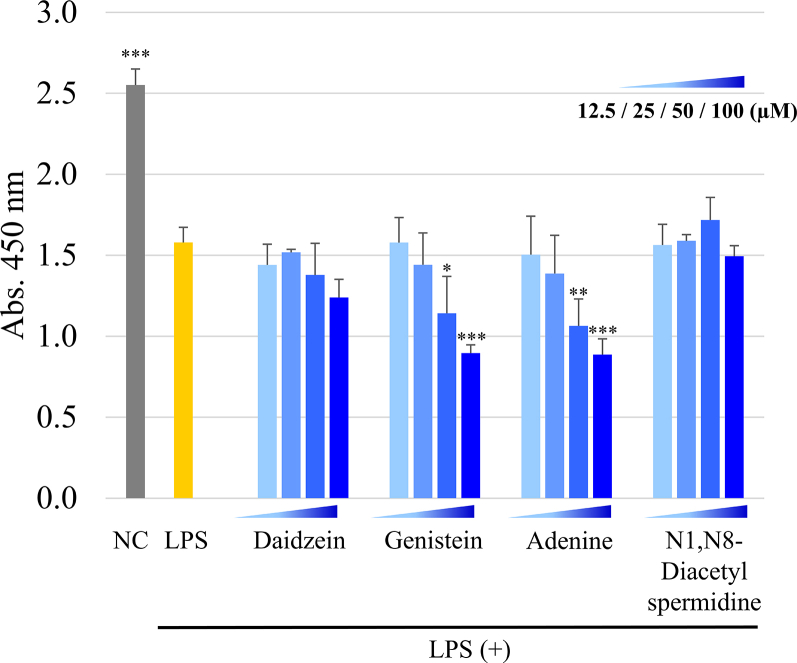


## Data Availability

Data will be made available on request.

## Abbreviations

SY: Soymilk yogurt (fermented soymilk)
HFD: High-fat diet
ND: Normal control diet
SM: Soymilk
BC: Bacterial cell of starter strain
LAB: Lactic acid bacteria
DMEM: Dulbecco's modified Eagle's medium
FBS: Fetal bovine serum
MRS: De Man, Rogosa, and Sharpe
NOR: Novel object recognition
RI: Recognition index
ELISA: Enzyme-linked immunosorbent assay
IL-6: Interleukin-6
TNF-α: Tumor Necrosis Factor-α
PBS: Phosphate-buffered saline
BSA: Bovine serum albumin
TBST: Tris-buffered saline containing Tween 20
PCR: Polymerase chain reaction
LC-MS/MS: Liquid chromatography-mass spectrometry/mass spectrometry
DIA: Data-independent acquisition
SYN: Synapsin
MBP: Myelin basic protein
MAP: Microtubule-associated protein
PCA: Principal component analysis
