# Supplementary material for: Soymilk yogurt prepared using Pediococcus pentosaceus TOKAI 759m ameliorates cognitive function through gut microbiota modulation in high-fat diet mice
Source: Curr Res Food Sci. 2025 Feb 3;10:100993. doi: 10.1016/j.crfs.2025.100993 (PMC11869912; doi:10.1016/j.crfs.2025.100993)
Supplement: Multimedia component 5 [file mmc5.docx]

**Supplementary Figures**


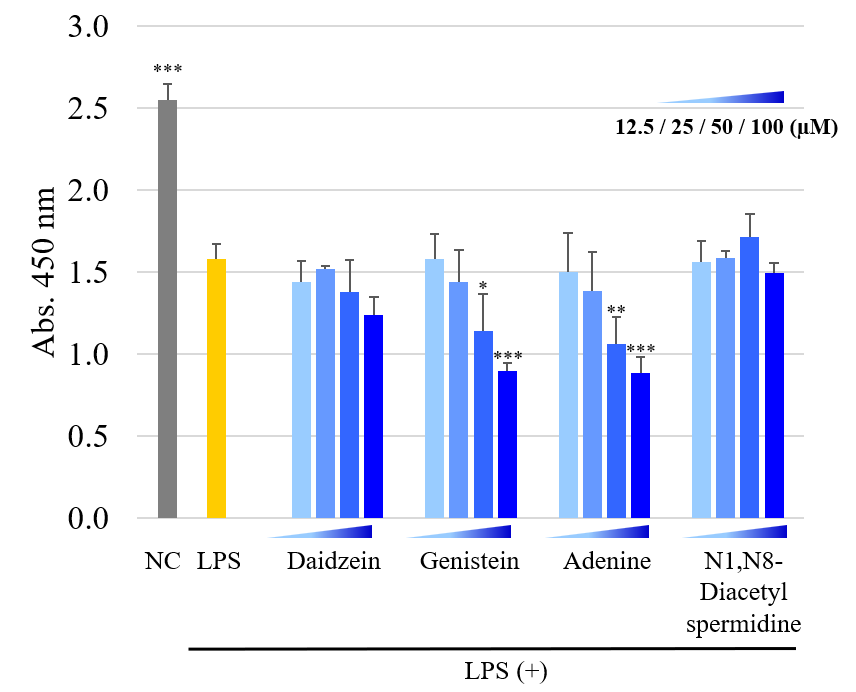


**Fig. S1.** Cell viability using components of SY in LPS-stimulated MG6 cells. Cells were seeded at 5×10^4^ cells/well in a 96-well plate and pre-incubated for 2 h. Then, the cells were treated with 12.5–100 μM of samples. After 24 h of incubation, the cells were stimulated with LPS (10 ng/mL) for 24 h. The cell viability of each concentration was analyzed using WST-8 assay. Results are expressed as the mean ± SD (n = 4). ^*^*P* < 0.05, ^**^*P* < 0.01, ^***^*P* < 0.001 vs. LPS group in the absence of samples. SY, soymilk yogurt (as fermented soymilk).
